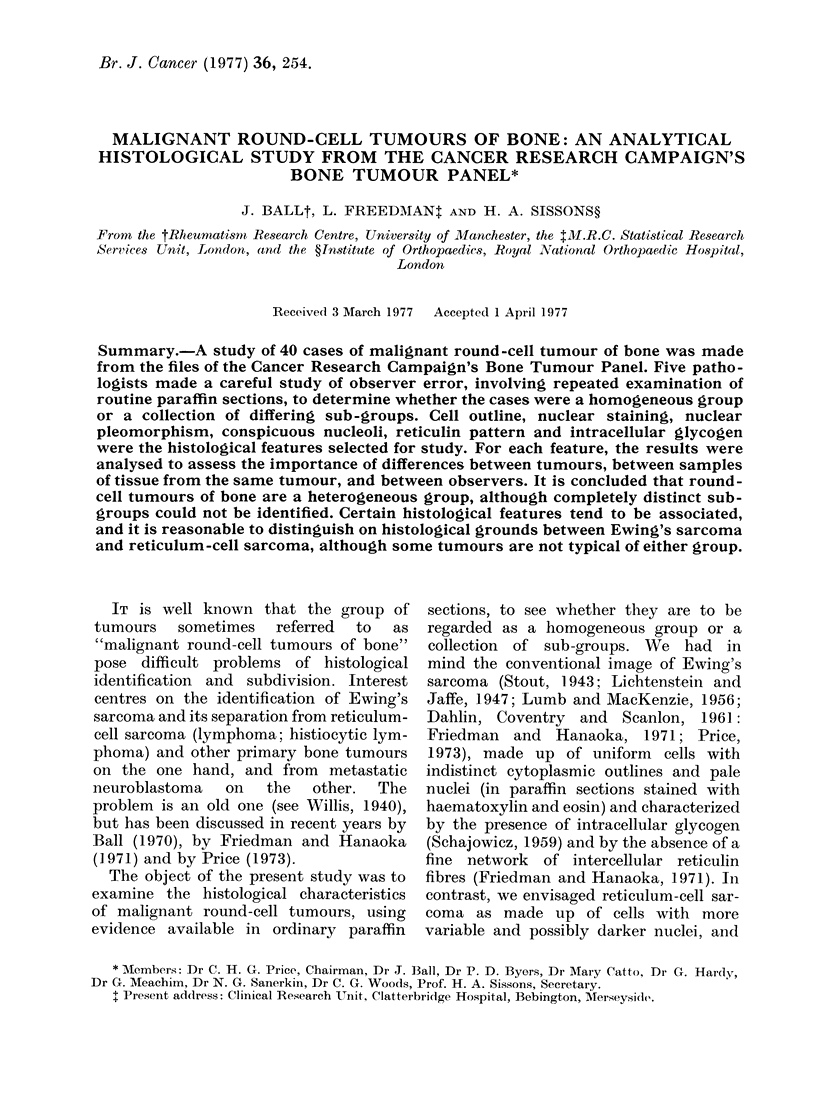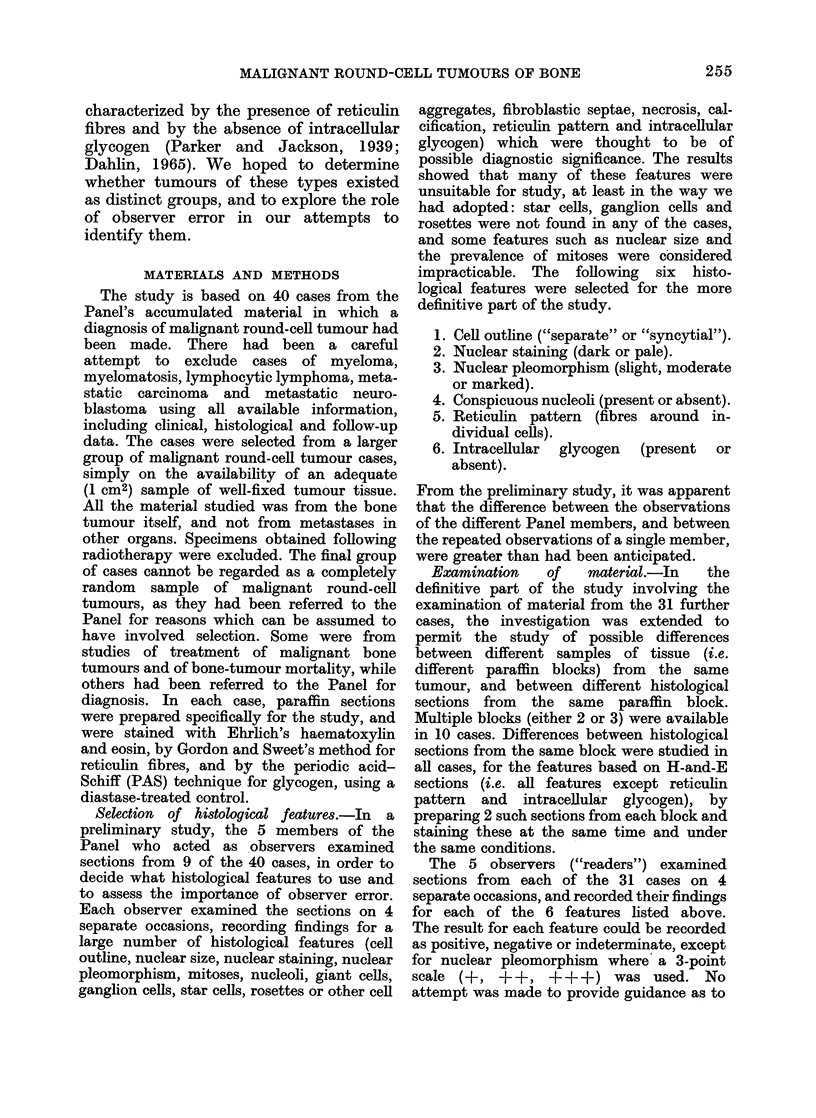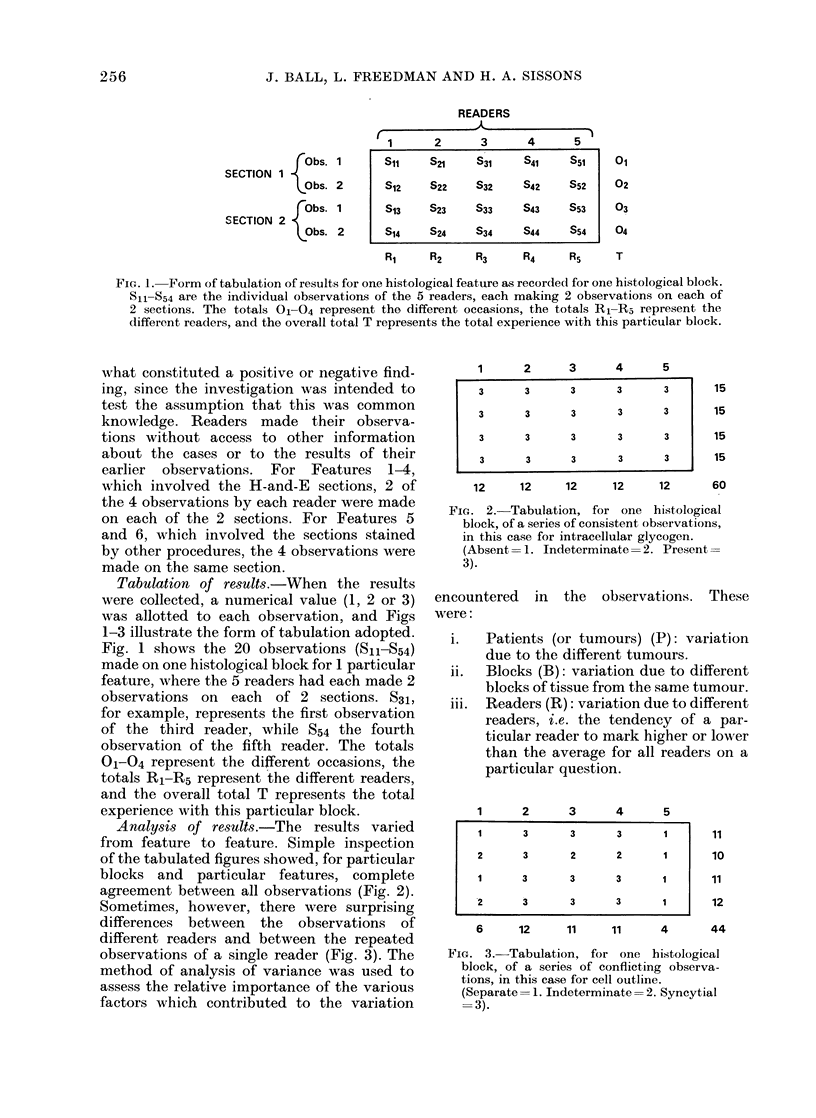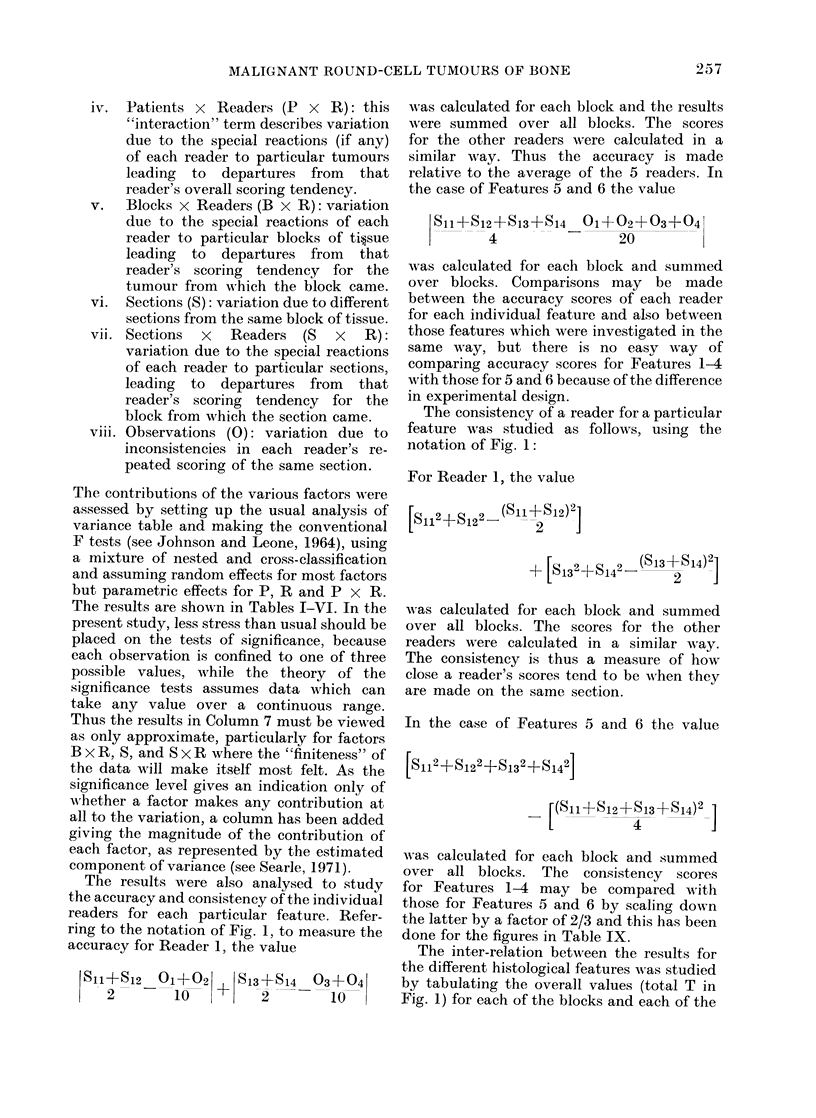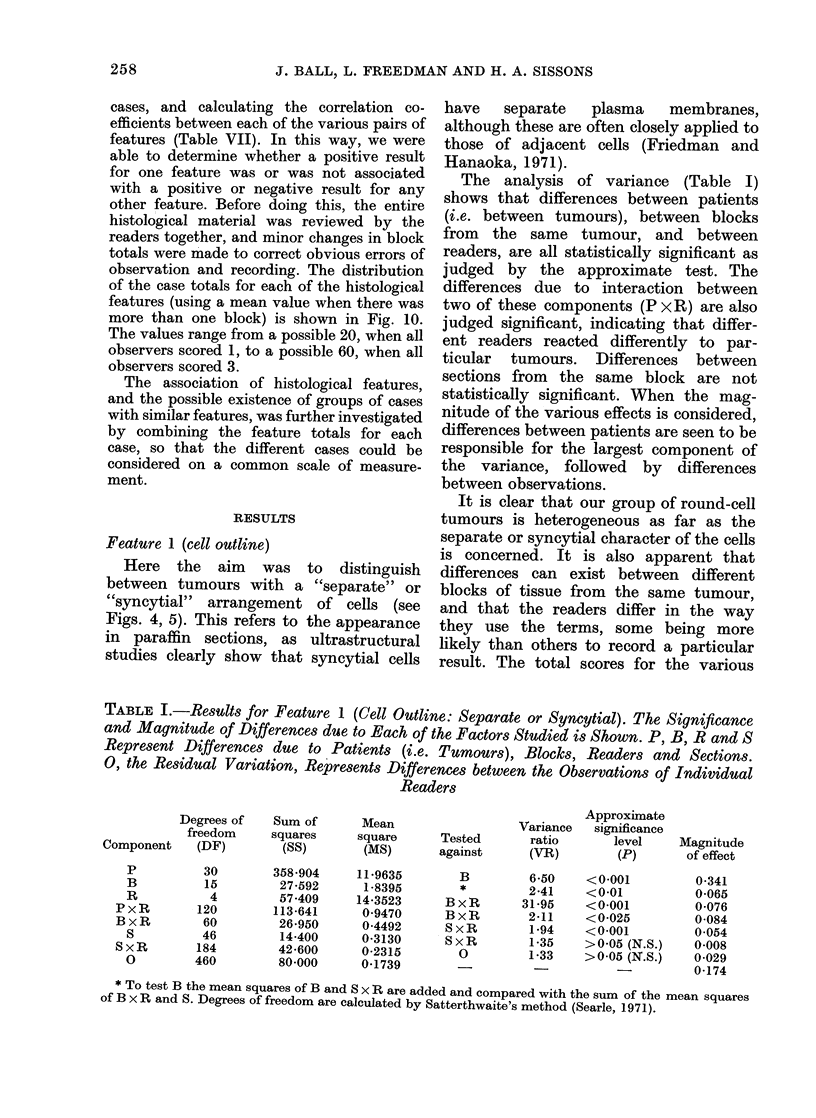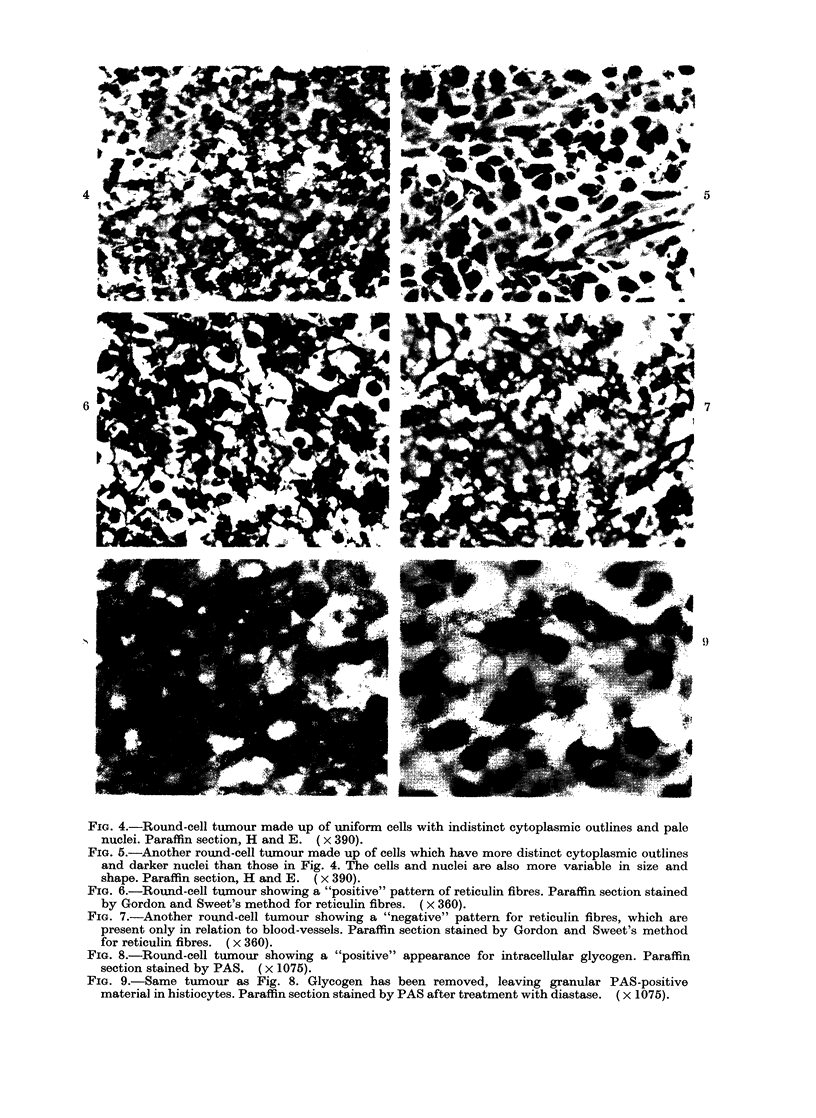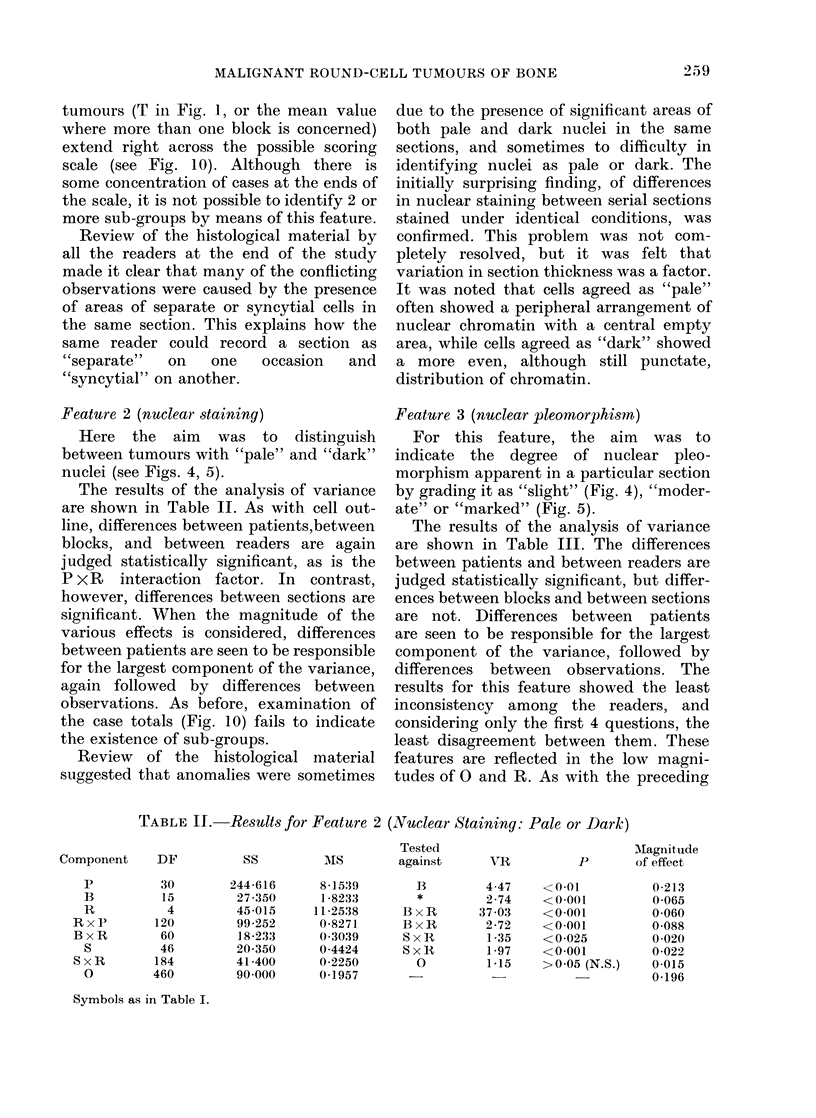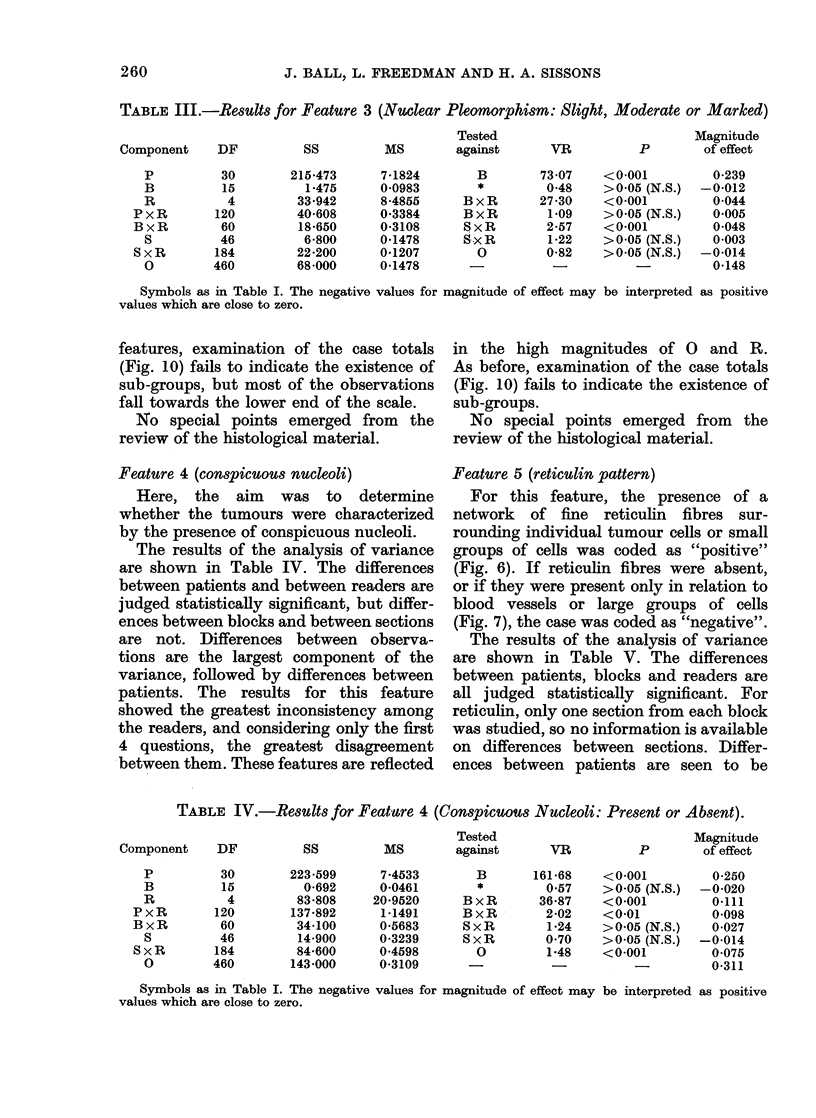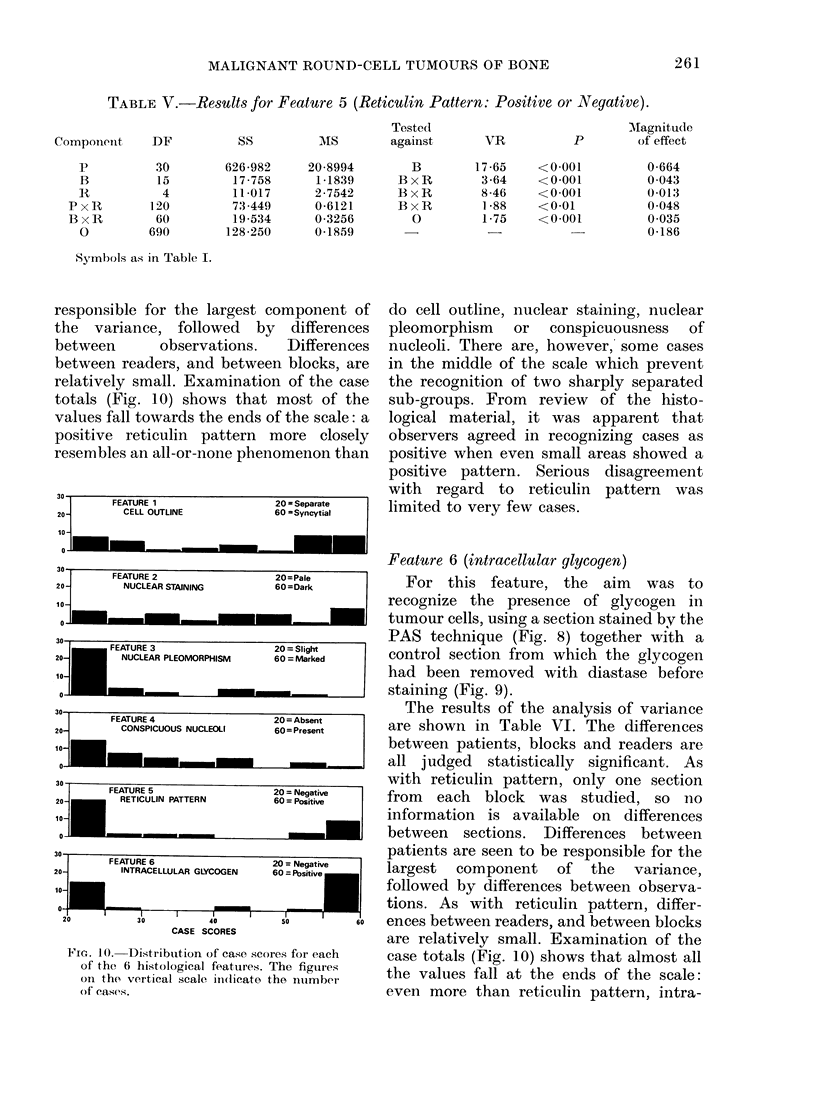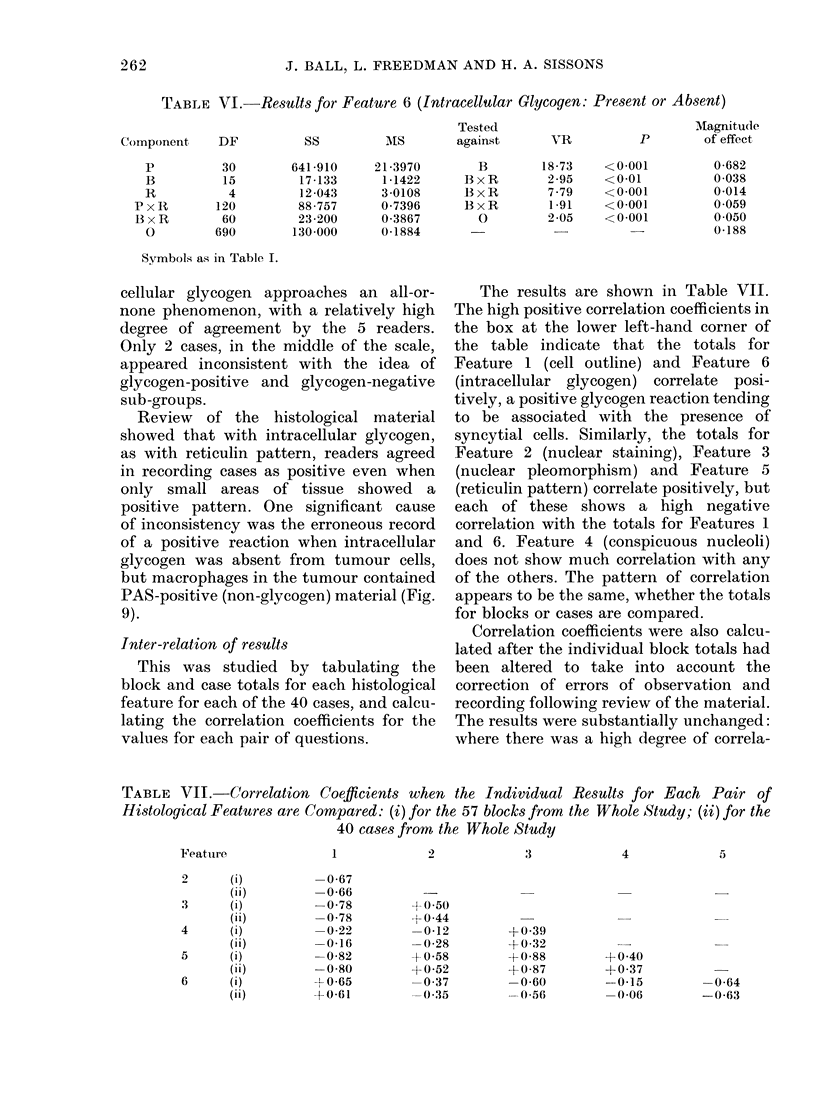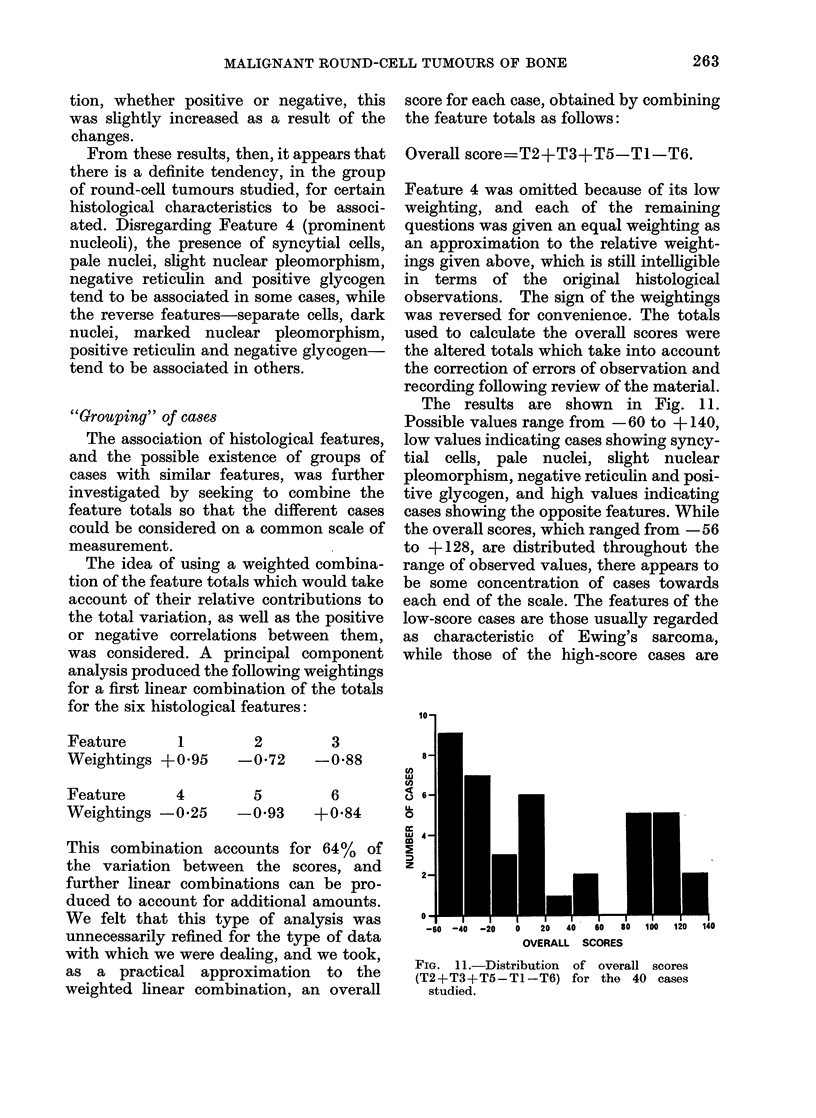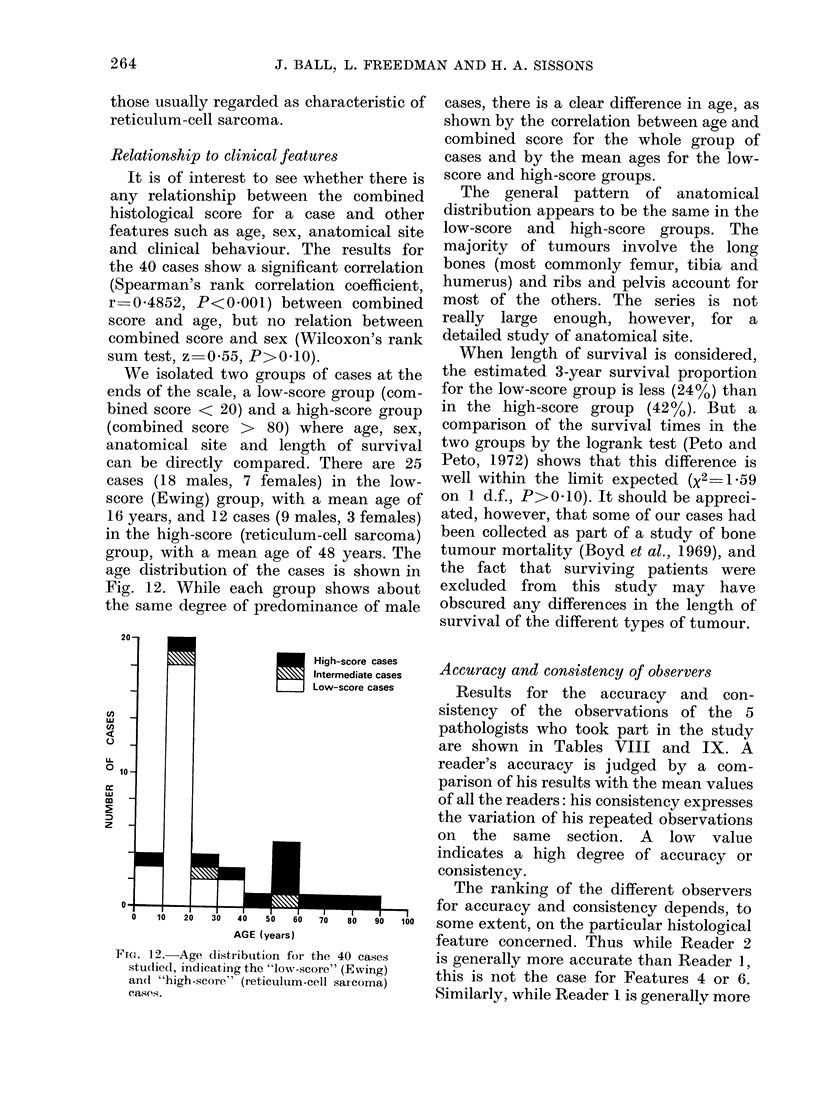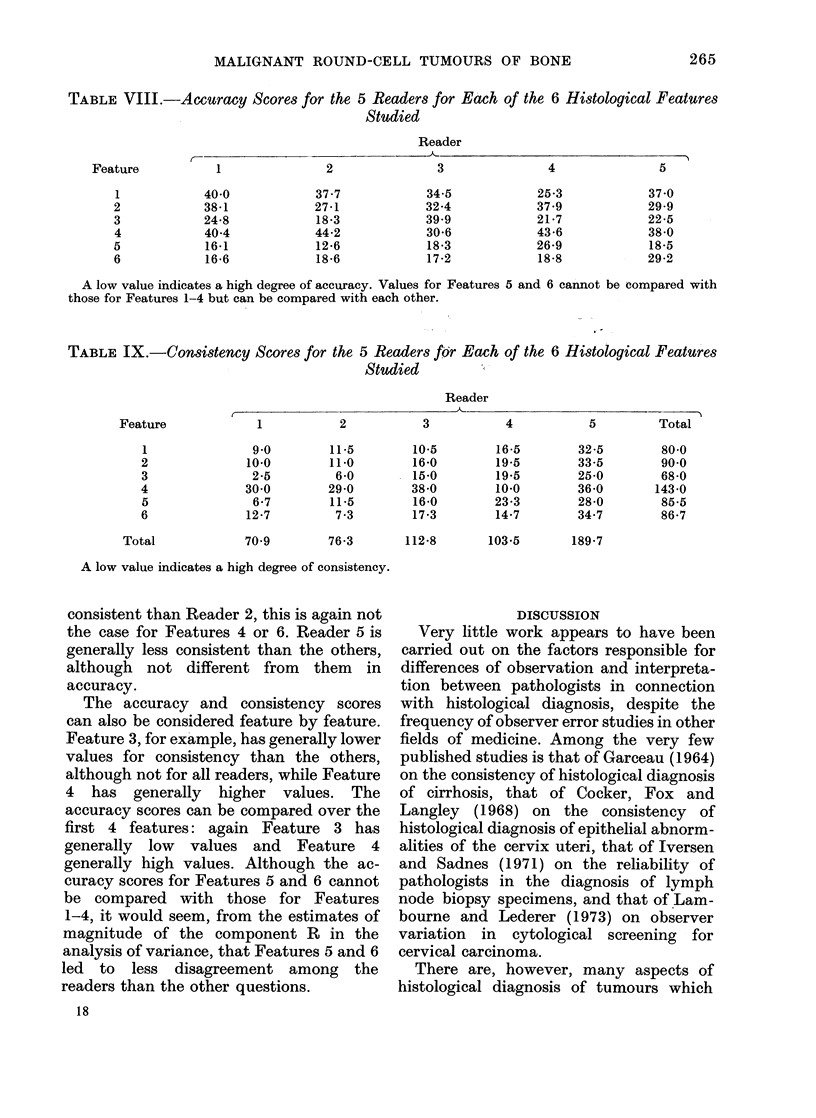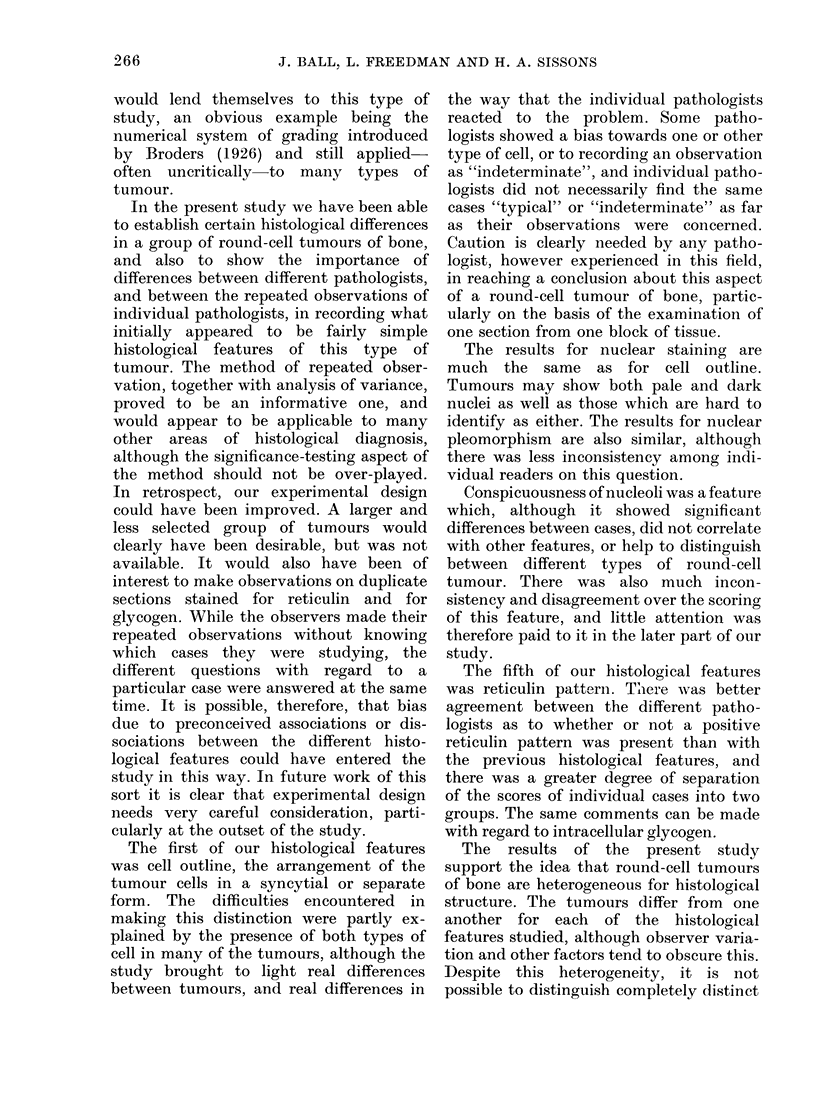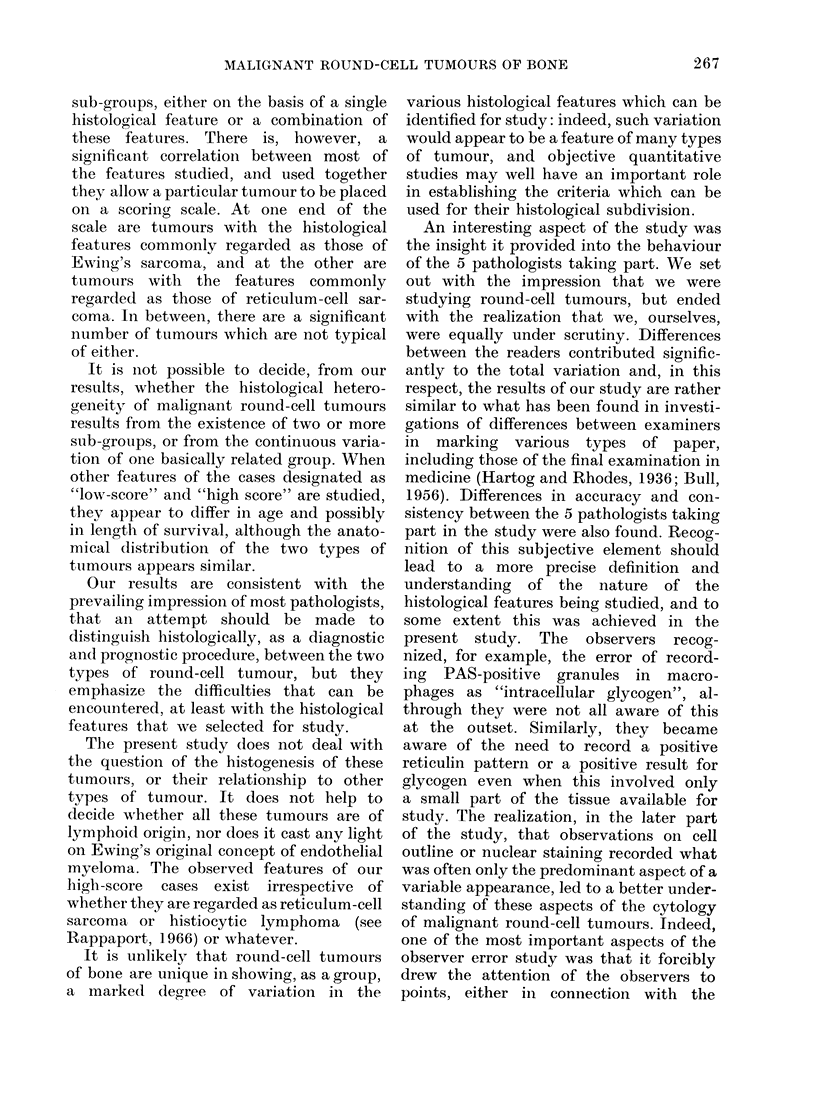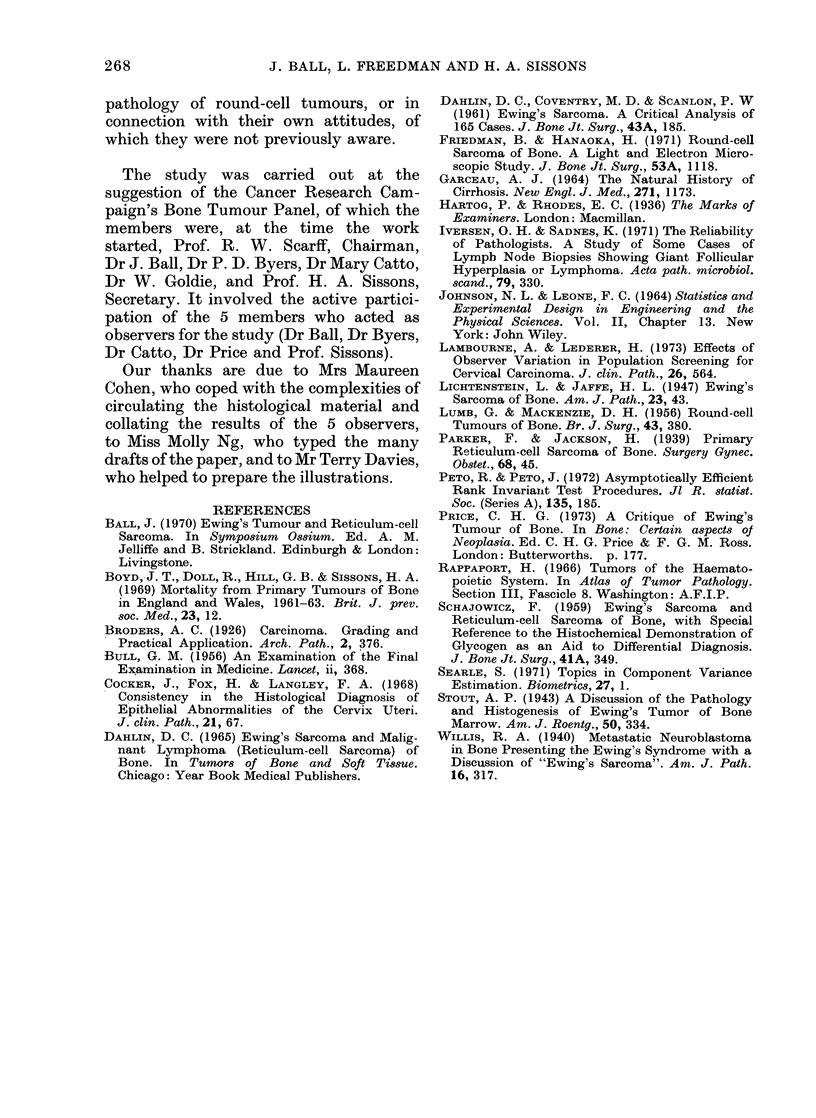# Malignant round-cell tumours of bone: an analytical histological study from the Cancer Research Campaign's bone tumour panel.

**DOI:** 10.1038/bjc.1977.185

**Published:** 1977-08

**Authors:** J. Ball, L. Freedman, H. A. Sissons

## Abstract

**Images:**


*Br. J. Cancer* (1977) **36**, 254.

### MALIGNANT ROUND-CELL TUMOURS OF BONE: AN ANALYTICAL HISTOLOGICAL STUDY FROM THE CANCER RESEARCH CAMPAIGN'S BONE TUMOUR PANEL*

J. BALL†, L. FREEDMAN‡ AND H. A. SISSONS§

*From the †Rheumatism Research Centre, University of Manchester, the ‡M.R.C. Statistical Research Services Unit, London, and the §Institute of Orthopaedics, Royal National Orthopaedic Hospital, London*

Received 3 March 1977 Accepted 1 April 1977

**Summary.—A study of 40 cases of malignant round-cell tumour of bone was made from the files of the Cancer Research Campaign's Bone Tumour Panel. Five pathologists made a careful study of observer error, involving repeated examination of routine paraffin sections, to determine whether the cases were a homogeneous group or a collection of differing sub-groups. Cell outline, nuclear staining, nuclear pleomorphism, conspicuous nucleoli, reticulin pattern and intracellular glycogen were the histological features selected for study. For each feature, the results were analysed to assess the importance of differences between tumours, between samples of tissue from the same tumour, and between observers. It is concluded that round-cell tumours of bone are a heterogeneous group, although completely distinct sub-groups could not be identified. Certain histological features tend to be associated, and it is reasonable to distinguish on histological grounds between Ewing's sarcoma and reticulum-cell sarcoma, although some tumours are not typical of either group.**

It is well known that the group of tumours sometimes referred to as "malignant round-cell tumours of bone" pose difficult problems of histological identification and subdivision. Interest centres on the identification of Ewing's sarcoma and its separation from reticulum-cell sarcoma (lymphoma; histiocytic lymphoma) and other primary bone tumours on the one hand, and from metastatic neuroblastoma on the other. The problem is an old one (see Willis, 1940), but has been discussed in recent years by Ball (1970), by Friedman and Hanaoka (1971) and by Price (1973).

The object of the present study was to examine the histological characteristics of malignant round-cell tumours, using evidence available in ordinary paraffin sections, to see whether they are to be regarded as a homogeneous group or a collection of sub-groups. We had in mind the conventional image of Ewing's sarcoma (Stout, 1943; Lichtenstein and Jaffe, 1947; Lumb and MacKenzie, 1956; Dahlin, Coventry and Scanlon, 1961: Friedman and Hanaoka, 1971; Price, 1973), made up of uniform cells with indistinct cytoplasmic outlines and pale nuclei (in paraffin sections stained with haematoxylin and eosin) and characterized by the presence of intracellular glycogen (Schajowicz, 1959) and by the absence of a fine network of intercellular reticulin fibres (Friedman and Hanaoka, 1971). In contrast, we envisaged reticulum-cell sarcoma as made up of cells with more variable and possibly darker nuclei, and

* Members: Dr C. H. G. Price, Chairman, Dr J. Ball, Dr P. D. Byers, Dr Mary Catto, Dr G. Hardy, Dr G. Meachim, Dr N. G. Sanerkin, Dr C. G. Woods, Prof. H. A. Sissons, Secretary.
‡ Present address: Clinical Research Unit, Clatterbridge Hospital, Bebington, Merseyside.

characterized by the presence of reticulin fibres and by the absence of intracellular glycogen (Parker and Jackson, 1939; Dahlin, 1965). We hoped to determine whether tumours of these types existed as distinct groups, and to explore the role of observer error in our attempts to identify them.

### MATERIALS AND METHODS

The study is based on 40 cases from the Panel's accumulated material in which a diagnosis of malignant round-cell tumour had been made. There had been a careful attempt to exclude cases of myeloma, myelomatosis, lymphocytic lymphoma, metastatic carcinoma and metastatic neuroblastoma using all available information, including clinical, histological and follow-up data. The cases were selected from a larger group of malignant round-cell tumour cases, simply on the availability of an adequate (1 $cm^2$) sample of well-fixed tumour tissue. All the material studied was from the bone tumour itself, and not from metastases in other organs. Specimens obtained following radiotherapy were excluded. The final group of cases cannot be regarded as a completely random sample of malignant round-cell tumours, as they had been referred to the Panel for reasons which can be assumed to have involved selection. Some were from studies of treatment of malignant bone tumours and of bone-tumour mortality, while others had been referred to the Panel for diagnosis. In each case, paraffin sections were prepared specifically for the study, and were stained with Ehrlich's haematoxylin and eosin, by Gordon and Sweet's method for reticulin fibres, and by the periodic acid–Schiff (PAS) technique for glycogen, using a diastase-treated control.

*Selection of histological features.*—In a preliminary study, the 5 members of the Panel who acted as observers examined sections from 9 of the 40 cases, in order to decide what histological features to use and to assess the importance of observer error. Each observer examined the sections on 4 separate occasions, recording findings for a large number of histological features (cell outline, nuclear size, nuclear staining, nuclear pleomorphism, mitoses, nucleoli, giant cells, ganglion cells, star cells, rosettes or other cell aggregates, fibroblastic septae, necrosis, calcification, reticulin pattern and intracellular glycogen) which were thought to be of possible diagnostic significance. The results showed that many of these features were unsuitable for study, at least in the way we had adopted: star cells, ganglion cells and rosettes were not found in any of the cases, and some features such as nuclear size and the prevalence of mitoses were considered impracticable. The following six histological features were selected for the more definitive part of the study.

1. Cell outline ("separate" or "syncytial").
2. Nuclear staining (dark or pale).
3. Nuclear pleomorphism (slight, moderate or marked).
4. Conspicuous nucleoli (present or absent).
5. Reticulin pattern (fibres around individual cells).
6. Intracellular glycogen (present or absent).

From the preliminary study, it was apparent that the difference between the observations of the different Panel members, and between the repeated observations of a single member, were greater than had been anticipated.

*Examination of material.*—In the definitive part of the study involving the examination of material from the 31 further cases, the investigation was extended to permit the study of possible differences between different samples of tissue (*i.e.* different paraffin blocks) from the same tumour, and between different histological sections from the same paraffin block. Multiple blocks (either 2 or 3) were available in 10 cases. Differences between histological sections from the same block were studied in all cases, for the features based on H-and-E sections (*i.e.* all features except reticulin pattern and intracellular glycogen), by preparing 2 such sections from each block and staining these at the same time and under the same conditions.

The 5 observers ("readers") examined sections from each of the 31 cases on 4 separate occasions, and recorded their findings for each of the 6 features listed above. The result for each feature could be recorded as positive, negative or indeterminate, except for nuclear pleomorphism where a 3-point scale (+, ++, +++) was used. No attempt was made to provide guidance as to

| | | READERS 1 | 2 | 3 | 4 | 5 | |
|---|---|---|---|---|---|---|---|
| SECTION 1 | Obs. 1 | $S_{11}$ | $S_{21}$ | $S_{31}$ | $S_{41}$ | $S_{51}$ | $O_1$ |
| | Obs. 2 | $S_{12}$ | $S_{22}$ | $S_{32}$ | $S_{42}$ | $S_{52}$ | $O_2$ |
| SECTION 2 | Obs. 1 | $S_{13}$ | $S_{23}$ | $S_{33}$ | $S_{43}$ | $S_{53}$ | $O_3$ |
| | Obs. 2 | $S_{14}$ | $S_{24}$ | $S_{34}$ | $S_{44}$ | $S_{54}$ | $O_4$ |
| | | $R_1$ | $R_2$ | $R_3$ | $R_4$ | $R_5$ | T |

FIG. 1.—Form of tabulation of results for one histological feature as recorded for one histological block. $S_{11}$–$S_{54}$ are the individual observations of the 5 readers, each making 2 observations on each of 2 sections. The totals $O_1$–$O_4$ represent the different occasions, the totals $R_1$–$R_5$ represent the different readers, and the overall total T represents the total experience with this particular block.

what constituted a positive or negative finding, since the investigation was intended to test the assumption that this was common knowledge. Readers made their observations without access to other information about the cases or to the results of their earlier observations. For Features 1–4, which involved the H-and-E sections, 2 of the 4 observations by each reader were made on each of the 2 sections. For Features 5 and 6, which involved the sections stained by other procedures, the 4 observations were made on the same section.

*Tabulation of results.*—When the results were collected, a numerical value (1, 2 or 3) was allotted to each observation, and Figs 1–3 illustrate the form of tabulation adopted. Fig. 1 shows the 20 observations ($S_{11}$–$S_{54}$) made on one histological block for 1 particular feature, where the 5 readers had each made 2 observations on each of 2 sections. $S_{31}$, for example, represents the first observation of the third reader, while $S_{54}$ the fourth observation of the fifth reader. The totals $O_1$–$O_4$ represent the different occasions, the totals $R_1$–$R_5$ represent the different readers, and the overall total T represents the total experience with this particular block.

*Analysis of results.*—The results varied from feature to feature. Simple inspection of the tabulated figures showed, for particular blocks and particular features, complete agreement between all observations (Fig. 2). Sometimes, however, there were surprising differences between the observations of different readers and between the repeated observations of a single reader (Fig. 3). The method of analysis of variance was used to assess the relative importance of the various factors which contributed to the variation

| 1 | 2 | 3 | 4 | 5 | |
|---|---|---|---|---|---|
| 3 | 3 | 3 | 3 | 3 | 15 |
| 3 | 3 | 3 | 3 | 3 | 15 |
| 3 | 3 | 3 | 3 | 3 | 15 |
| 3 | 3 | 3 | 3 | 3 | 15 |
| 12 | 12 | 12 | 12 | 12 | 60 |

FIG. 2.—Tabulation, for one histological block, of a series of consistent observations, in this case for intracellular glycogen. (Absent = 1. Indeterminate = 2. Present = 3).

encountered in the observations. These were:

i. Patients (or tumours) (P): variation due to the different tumours.
ii. Blocks (B): variation due to different blocks of tissue from the same tumour.
iii. Readers (R): variation due to different readers, *i.e.* the tendency of a particular reader to mark higher or lower than the average for all readers on a particular question.

| 1 | 2 | 3 | 4 | 5 | |
|---|---|---|---|---|---|
| 1 | 3 | 3 | 3 | 1 | 11 |
| 2 | 3 | 2 | 2 | 1 | 10 |
| 1 | 3 | 3 | 3 | 1 | 11 |
| 2 | 3 | 3 | 3 | 1 | 12 |
| 6 | 12 | 11 | 11 | 4 | 44 |

FIG. 3.—Tabulation, for one histological block, of a series of conflicting observations, in this case for cell outline. (Separate = 1. Indeterminate = 2. Syncytial = 3).

iv. Patients × Readers (P × R): this "interaction" term describes variation due to the special reactions (if any) of each reader to particular tumours leading to departures from that reader's overall scoring tendency.
v. Blocks × Readers (B × R): variation due to the special reactions of each reader to particular blocks of tissue leading to departures from that reader's scoring tendency for the tumour from which the block came.
vi. Sections (S): variation due to different sections from the same block of tissue.
vii. Sections × Readers (S × R): variation due to the special reactions of each reader to particular sections, leading to departures from that reader's scoring tendency for the block from which the section came.
viii. Observations (O): variation due to inconsistencies in each reader's repeated scoring of the same section.

The contributions of the various factors were assessed by setting up the usual analysis of variance table and making the conventional F tests (see Johnson and Leone, 1964), using a mixture of nested and cross-classification and assuming random effects for most factors but parametric effects for P, R and P × R. The results are shown in Tables I–VI. In the present study, less stress than usual should be placed on the tests of significance, because each observation is confined to one of three possible values, while the theory of the significance tests assumes data which can take any value over a continuous range. Thus the results in Column 7 must be viewed as only approximate, particularly for factors B×R, S, and S×R where the "finiteness" of the data will make itself most felt. As the significance level gives an indication only of whether a factor makes any contribution at all to the variation, a column has been added giving the magnitude of the contribution of each factor, as represented by the estimated component of variance (see Searle, 1971).

The results were also analysed to study the accuracy and consistency of the individual readers for each particular feature. Referring to the notation of Fig. 1, to measure the accuracy for Reader 1, the value

$$\left|\frac{S_{11}+S_{12}}{2}-\frac{O_1+O_2}{10}\right|+\left|\frac{S_{13}+S_{14}}{2}-\frac{O_3+O_4}{10}\right|$$

was calculated for each block and the results were summed over all blocks. The scores for the other readers were calculated in a similar way. Thus the accuracy is made relative to the average of the 5 readers. In the case of Features 5 and 6 the value

$$\left|\frac{S_{11}+S_{12}+S_{13}+S_{14}}{4}-\frac{O_1+O_2+O_3+O_4}{20}\right|$$

was calculated for each block and summed over blocks. Comparisons may be made between the accuracy scores of each reader for each individual feature and also between those features which were investigated in the same way, but there is no easy way of comparing accuracy scores for Features 1–4 with those for 5 and 6 because of the difference in experimental design.

The consistency of a reader for a particular feature was studied as follows, using the notation of Fig. 1:

For Reader 1, the value

$$\left[S_{11}^2+S_{12}^2-\frac{(S_{11}+S_{12})^2}{2}\right]+\left[S_{13}^2+S_{14}^2-\frac{(S_{13}+S_{14})^2}{2}\right]$$

was calculated for each block and summed over all blocks. The scores for the other readers were calculated in a similar way. The consistency is thus a measure of how close a reader's scores tend to be when they are made on the same section.

In the case of Features 5 and 6 the value

$$\left[S_{11}^2+S_{12}^2+S_{13}^2+S_{14}^2\right]-\left[\frac{(S_{11}+S_{12}+S_{13}+S_{14})^2}{4}\right]$$

was calculated for each block and summed over all blocks. The consistency scores for Features 1–4 may be compared with those for Features 5 and 6 by scaling down the latter by a factor of 2/3 and this has been done for the figures in Table IX.

The inter-relation between the results for the different histological features was studied by tabulating the overall values (total T in Fig. 1) for each of the blocks and each of the cases, and calculating the correlation coefficients between each of the various pairs of features (Table VII). In this way, we were able to determine whether a positive result for one feature was or was not associated with a positive or negative result for any other feature. Before doing this, the entire histological material was reviewed by the readers together, and minor changes in block totals were made to correct obvious errors of observation and recording. The distribution of the case totals for each of the histological features (using a mean value when there was more than one block) is shown in Fig. 10. The values range from a possible 20, when all observers scored 1, to a possible 60, when all observers scored 3.

The association of histological features, and the possible existence of groups of cases with similar features, was further investigated by combining the feature totals for each case, so that the different cases could be considered on a common scale of measurement.

### RESULTS

*Feature* 1 (*cell outline*)

Here the aim was to distinguish between tumours with a "separate" or "syncytial" arrangement of cells (see Figs. 4, 5). This refers to the appearance in paraffin sections, as ultrastructural studies clearly show that syncytial cells have separate plasma membranes, although these are often closely applied to those of adjacent cells (Friedman and Hanaoka, 1971).

The analysis of variance (Table I) shows that differences between patients (*i.e.* between tumours), between blocks from the same tumour, and between readers, are all statistically significant as judged by the approximate test. The differences due to interaction between two of these components (P×R) are also judged significant, indicating that different readers reacted differently to particular tumours. Differences between sections from the same block are not statistically significant. When the magnitude of the various effects is considered, differences between patients are seen to be responsible for the largest component of the variance, followed by differences between observations.

It is clear that our group of round-cell tumours is heterogeneous as far as the separate or syncytial character of the cells is concerned. It is also apparent that differences can exist between different blocks of tissue from the same tumour, and that the readers differ in the way they use the terms, some being more likely than others to record a particular result. The total scores for the various

TABLE I.—*Results for Feature* 1 (*Cell Outline: Separate or Syncytial*). *The Significance and Magnitude of Differences due to Each of the Factors Studied is Shown. P, B, R and S Represent Differences due to Patients* (*i.e. Tumours*), *Blocks, Readers and Sections. O, the Residual Variation, Represents Differences between the Observations of Individual Readers*

| Component | Degrees of freedom (DF) | Sum of squares (SS) | Mean square (MS) | Tested against | Variance ratio (VR) | Approximate significance level (*P*) | Magnitude of effect |
|---|---|---|---|---|---|---|---|
| P | 30 | 358·904 | 11·9635 | B | 6·50 | <0·001 | 0·341 |
| B | 15 | 27·592 | 1·8395 | * | 2·41 | <0·01 | 0·065 |
| R | 4 | 57·409 | 14·3523 | B×R | 31·95 | <0·001 | 0·076 |
| P×R | 120 | 113·641 | 0·9470 | B×R | 2·11 | <0·025 | 0·084 |
| B×R | 60 | 26·950 | 0·4492 | S×R | 1·94 | <0·001 | 0·054 |
| S | 46 | 14·400 | 0·3130 | S×R | 1·35 | >0·05 (N.S.) | 0·008 |
| S×R | 184 | 42·600 | 0·2315 | O | 1·33 | >0·05 (N.S.) | 0·029 |
| O | 460 | 80·000 | 0·1739 | — | — | — | 0·174 |

* To test B the mean squares of B and S×R are added and compared with the sum of the mean squares of B×R and S. Degrees of freedom are calculated by Satterthwaite's method (Searle, 1971).

4

5

6

7

8

9

FIG. 4.—Round-cell tumour made up of uniform cells with indistinct cytoplasmic outlines and pale nuclei. Paraffin section, H and E. (× 390).

FIG. 5.—Another round-cell tumour made up of cells which have more distinct cytoplasmic outlines and darker nuclei than those in Fig. 4. The cells and nuclei are also more variable in size and shape. Paraffin section, H and E. (× 390).

FIG. 6.—Round-cell tumour showing a "positive" pattern of reticulin fibres. Paraffin section stained by Gordon and Sweet's method for reticulin fibres. (× 360).

FIG. 7.—Another round-cell tumour showing a "negative" pattern for reticulin fibres, which are present only in relation to blood-vessels. Paraffin section stained by Gordon and Sweet's method for reticulin fibres. (× 360).

FIG. 8.—Round-cell tumour showing a "positive" appearance for intracellular glycogen. Paraffin section stained by PAS. (× 1075).

FIG. 9.—Same tumour as Fig. 8. Glycogen has been removed, leaving granular PAS-positive material in histiocytes. Paraffin section stained by PAS after treatment with diastase. (× 1075).

tumours (T in Fig. 1, or the mean value where more than one block is concerned) extend right across the possible scoring scale (see Fig. 10). Although there is some concentration of cases at the ends of the scale, it is not possible to identify 2 or more sub-groups by means of this feature.

Review of the histological material by all the readers at the end of the study made it clear that many of the conflicting observations were caused by the presence of areas of separate or syncytial cells in the same section. This explains how the same reader could record a section as "separate" on one occasion and "syncytial" on another.

*Feature* 2 (*nuclear staining*)

Here the aim was to distinguish between tumours with "pale" and "dark" nuclei (see Figs. 4, 5).

The results of the analysis of variance are shown in Table II. As with cell outline, differences between patients,between blocks, and between readers are again judged statistically significant, as is the P×R interaction factor. In contrast, however, differences between sections are significant. When the magnitude of the various effects is considered, differences between patients are seen to be responsible for the largest component of the variance, again followed by differences between observations. As before, examination of the case totals (Fig. 10) fails to indicate the existence of sub-groups.

Review of the histological material suggested that anomalies were sometimes due to the presence of significant areas of both pale and dark nuclei in the same sections, and sometimes to difficulty in identifying nuclei as pale or dark. The initially surprising finding, of differences in nuclear staining between serial sections stained under identical conditions, was confirmed. This problem was not completely resolved, but it was felt that variation in section thickness was a factor. It was noted that cells agreed as "pale" often showed a peripheral arrangement of nuclear chromatin with a central empty area, while cells agreed as "dark" showed a more even, although still punctate, distribution of chromatin.

*Feature* 3 (*nuclear pleomorphism*)

For this feature, the aim was to indicate the degree of nuclear pleomorphism apparent in a particular section by grading it as "slight" (Fig. 4), "moderate" or "marked" (Fig. 5).

The results of the analysis of variance are shown in Table III. The differences between patients and between readers are judged statistically significant, but differences between blocks and between sections are not. Differences between patients are seen to be responsible for the largest component of the variance, followed by differences between observations. The results for this feature showed the least inconsistency among the readers, and considering only the first 4 questions, the least disagreement between them. These features are reflected in the low magnitudes of O and R. As with the preceding

TABLE II.—*Results for Feature* 2 (*Nuclear Staining: Pale or Dark*)

| Component | DF | SS | MS | Tested against | VR | *P* | Magnitude of effect |
|---|---|---|---|---|---|---|---|
| P | 30 | 244·616 | 8·1539 | B | 4·47 | <0·01 | 0·213 |
| B | 15 | 27·350 | 1·8233 | * | 2·74 | <0·001 | 0·065 |
| R | 4 | 45·015 | 11·2538 | B×R | 37·03 | <0·001 | 0·060 |
| R×P | 120 | 99·252 | 0·8271 | B×R | 2·72 | <0·001 | 0·088 |
| B×R | 60 | 18·233 | 0·3039 | S×R | 1·35 | <0·025 | 0·020 |
| S | 46 | 20·350 | 0·4424 | S×R | 1·97 | <0·001 | 0·022 |
| S×R | 184 | 41·400 | 0·2250 | O | 1·15 | >0·05 (N.S.) | 0·015 |
| O | 460 | 90·000 | 0·1957 | — | — | — | 0·196 |

Symbols as in Table I.

TABLE III.—*Results for Feature* 3 (*Nuclear Pleomorphism: Slight, Moderate or Marked*)

| Component | DF | SS | MS | Tested against | VR | *P* | Magnitude of effect |
|---|---|---|---|---|---|---|---|
| P | 30 | 215·473 | 7·1824 | B | 73·07 | <0·001 | 0·239 |
| B | 15 | 1·475 | 0·0983 | * | 0·48 | >0·05 (N.S.) | −0·012 |
| R | 4 | 33·942 | 8·4855 | B×R | 27·30 | <0·001 | 0·044 |
| P×R | 120 | 40·608 | 0·3384 | B×R | 1·09 | >0·05 (N.S.) | 0·005 |
| B×R | 60 | 18·650 | 0·3108 | S×R | 2·57 | <0·001 | 0·048 |
| S | 46 | 6·800 | 0·1478 | S×R | 1·22 | >0·05 (N.S.) | 0·003 |
| S×R | 184 | 22·200 | 0·1207 | O | 0·82 | >0·05 (N.S.) | −0·014 |
| O | 460 | 68·000 | 0·1478 | — | — | — | 0·148 |

Symbols as in Table I. The negative values for magnitude of effect may be interpreted as positive values which are close to zero.

features, examination of the case totals (Fig. 10) fails to indicate the existence of sub-groups, but most of the observations fall towards the lower end of the scale.

No special points emerged from the review of the histological material.

*Feature* 4 (*conspicuous nucleoli*)

Here, the aim was to determine whether the tumours were characterized by the presence of conspicuous nucleoli.

The results of the analysis of variance are shown in Table IV. The differences between patients and between readers are judged statistically significant, but differences between blocks and between sections are not. Differences between observations are the largest component of the variance, followed by differences between patients. The results for this feature showed the greatest inconsistency among the readers, and considering only the first 4 questions, the greatest disagreement between them. These features are reflected in the high magnitudes of O and R. As before, examination of the case totals (Fig. 10) fails to indicate the existence of sub-groups.

No special points emerged from the review of the histological material.

*Feature* 5 (*reticulin pattern*)

For this feature, the presence of a network of fine reticulin fibres surrounding individual tumour cells or small groups of cells was coded as "positive" (Fig. 6). If reticulin fibres were absent, or if they were present only in relation to blood vessels or large groups of cells (Fig. 7), the case was coded as "negative".

The results of the analysis of variance are shown in Table V. The differences between patients, blocks and readers are all judged statistically significant. For reticulin, only one section from each block was studied, so no information is available on differences between sections. Differences between patients are seen to be

TABLE IV.—*Results for Feature* 4 (*Conspicuous Nucleoli: Present or Absent*).

| Component | DF | SS | MS | Tested against | VR | *P* | Magnitude of effect |
|---|---|---|---|---|---|---|---|
| P | 30 | 223·599 | 7·4533 | B | 161·68 | <0·001 | 0·250 |
| B | 15 | 0·692 | 0·0461 | * | 0·57 | >0·05 (N.S.) | −0·020 |
| R | 4 | 83·808 | 20·9520 | B×R | 36·87 | <0·001 | 0·111 |
| P×R | 120 | 137·892 | 1·1491 | B×R | 2·02 | <0·01 | 0·098 |
| B×R | 60 | 34·100 | 0·5683 | S×R | 1·24 | >0·05 (N.S.) | 0·027 |
| S | 46 | 14·900 | 0·3239 | S×R | 0·70 | >0·05 (N.S.) | −0·014 |
| S×R | 184 | 84·600 | 0·4598 | O | 1·48 | <0·001 | 0·075 |
| O | 460 | 143·000 | 0·3109 | — | — | — | 0·311 |

Symbols as in Table I. The negative values for magnitude of effect may be interpreted as positive values which are close to zero.

TABLE V.—*Results for Feature* 5 (*Reticulin Pattern: Positive or Negative*).

| Component | DF | SS | MS | Tested against | VR | *P* | Magnitude of effect |
|---|---|---|---|---|---|---|---|
| P | 30 | 626·982 | 20·8994 | B | 17·65 | <0·001 | 0·664 |
| B | 15 | 17·758 | 1·1839 | B×R | 3·64 | <0·001 | 0·043 |
| R | 4 | 11·017 | 2·7542 | B×R | 8·46 | <0·001 | 0·013 |
| P×R | 120 | 73·449 | 0·6121 | B×R | 1·88 | <0·01 | 0·048 |
| B×R | 60 | 19·534 | 0·3256 | O | 1·75 | <0·001 | 0·035 |
| O | 690 | 128·250 | 0·1859 | — | — | — | 0·186 |

Symbols as in Table I.

responsible for the largest component of the variance, followed by differences between observations. Differences between readers, and between blocks, are relatively small. Examination of the case totals (Fig. 10) shows that most of the values fall towards the ends of the scale: a positive reticulin pattern more closely resembles an all-or-none phenomenon than do cell outline, nuclear staining, nuclear pleomorphism or conspicuousness of nucleoli. There are, however, some cases in the middle of the scale which prevent the recognition of two sharply separated sub-groups. From review of the histological material, it was apparent that observers agreed in recognizing cases as positive when even small areas showed a positive pattern. Serious disagreement with regard to reticulin pattern was limited to very few cases.

FIG. 10.—Distribution of case scores for each of the 6 histological features. The figures on the vertical scale indicate the number of cases.

*Feature* 6 (*intracellular glycogen*)

For this feature, the aim was to recognize the presence of glycogen in tumour cells, using a section stained by the PAS technique (Fig. 8) together with a control section from which the glycogen had been removed with diastase before staining (Fig. 9).

The results of the analysis of variance are shown in Table VI. The differences between patients, blocks and readers are all judged statistically significant. As with reticulin pattern, only one section from each block was studied, so no information is available on differences between sections. Differences between patients are seen to be responsible for the largest component of the variance, followed by differences between observations. As with reticulin pattern, differences between readers, and between blocks are relatively small. Examination of the case totals (Fig. 10) shows that almost all the values fall at the ends of the scale: even more than reticulin pattern, intra-

TABLE VI.—*Results for Feature 6 (Intracellular Glycogen: Present or Absent)*

| Component | DF | SS | MS | Tested against | VR | *P* | Magnitude of effect |
|---|---|---|---|---|---|---|---|
| P | 30 | 641·910 | 21·3970 | B | 18·73 | <0·001 | 0·682 |
| B | 15 | 17·133 | 1·1422 | B×R | 2·95 | <0·01 | 0·038 |
| R | 4 | 12·043 | 3·0108 | B×R | 7·79 | <0·001 | 0·014 |
| P×R | 120 | 88·757 | 0·7396 | B×R | 1·91 | <0·001 | 0·059 |
| B×R | 60 | 23·200 | 0·3867 | O | 2·05 | <0·001 | 0·050 |
| O | 690 | 130·000 | 0·1884 | — | — | — | 0·188 |

Symbols as in Table I.

cellular glycogen approaches an all-or-none phenomenon, with a relatively high degree of agreement by the 5 readers. Only 2 cases, in the middle of the scale, appeared inconsistent with the idea of glycogen-positive and glycogen-negative sub-groups.

Review of the histological material showed that with intracellular glycogen, as with reticulin pattern, readers agreed in recording cases as positive even when only small areas of tissue showed a positive pattern. One significant cause of inconsistency was the erroneous record of a positive reaction when intracellular glycogen was absent from tumour cells, but macrophages in the tumour contained PAS-positive (non-glycogen) material (Fig. 9).

*Inter-relation of results*

This was studied by tabulating the block and case totals for each histological feature for each of the 40 cases, and calculating the correlation coefficients for the values for each pair of questions.

The results are shown in Table VII. The high positive correlation coefficients in the box at the lower left-hand corner of the table indicate that the totals for Feature 1 (cell outline) and Feature 6 (intracellular glycogen) correlate positively, a positive glycogen reaction tending to be associated with the presence of syncytial cells. Similarly, the totals for Feature 2 (nuclear staining), Feature 3 (nuclear pleomorphism) and Feature 5 (reticulin pattern) correlate positively, but each of these shows a high negative correlation with the totals for Features 1 and 6. Feature 4 (conspicuous nucleoli) does not show much correlation with any of the others. The pattern of correlation appears to be the same, whether the totals for blocks or cases are compared.

Correlation coefficients were also calculated after the individual block totals had been altered to take into account the correction of errors of observation and recording following review of the material. The results were substantially unchanged: where there was a high degree of correla-

TABLE VII.—*Correlation Coefficients when the Individual Results for Each Pair of Histological Features are Compared: (i) for the 57 blocks from the Whole Study; (ii) for the 40 cases from the Whole Study*

| Feature | | 1 | 2 | 3 | 4 | 5 |
|---|---|---|---|---|---|---|
| 2 | (i) | −0·67 | | | | |
| | (ii) | −0·66 | — | — | — | — |
| 3 | (i) | −0·78 | +0·50 | | | |
| | (ii) | −0·78 | +0·44 | — | — | — |
| 4 | (i) | −0·22 | −0·12 | +0·39 | | |
| | (ii) | −0·16 | −0·28 | +0·32 | — | — |
| 5 | (i) | −0·82 | +0·58 | +0·88 | +0·40 | |
| | (ii) | −0·80 | +0·52 | +0·87 | +0·37 | — |
| 6 | (i) | +0·65 | −0·37 | −0·60 | −0·15 | −0·64 |
| | (ii) | +0·61 | −0·35 | −0·56 | −0·06 | −0·63 |

tion, whether positive or negative, this was slightly increased as a result of the changes.

From these results, then, it appears that there is a definite tendency, in the group of round-cell tumours studied, for certain histological characteristics to be associated. Disregarding Feature 4 (prominent nucleoli), the presence of syncytial cells, pale nuclei, slight nuclear pleomorphism, negative reticulin and positive glycogen tend to be associated in some cases, while the reverse features—separate cells, dark nuclei, marked nuclear pleomorphism, positive reticulin and negative glycogen—tend to be associated in others.

*"Grouping" of cases*

The association of histological features, and the possible existence of groups of cases with similar features, was further investigated by seeking to combine the feature totals so that the different cases could be considered on a common scale of measurement.

The idea of using a weighted combination of the feature totals which would take account of their relative contributions to the total variation, as well as the positive or negative correlations between them, was considered. A principal component analysis produced the following weightings for a first linear combination of the totals for the six histological features:

| Feature | 1 | 2 | 3 |
|---|---|---|---|
| Weightings | +0·95 | −0·72 | −0·88 |

| Feature | 4 | 5 | 6 |
|---|---|---|---|
| Weightings | −0·25 | −0·93 | +0·84 |

This combination accounts for 64% of the variation between the scores, and further linear combinations can be produced to account for additional amounts. We felt that this type of analysis was unnecessarily refined for the type of data with which we were dealing, and we took, as a practical approximation to the weighted linear combination, an overall score for each case, obtained by combining the feature totals as follows:

$$\text{Overall score} = T2 + T3 + T5 - T1 - T6.$$

Feature 4 was omitted because of its low weighting, and each of the remaining questions was given an equal weighting as an approximation to the relative weightings given above, which is still intelligible in terms of the original histological observations. The sign of the weightings was reversed for convenience. The totals used to calculate the overall scores were the altered totals which take into account the correction of errors of observation and recording following review of the material.

The results are shown in Fig. 11. Possible values range from −60 to +140, low values indicating cases showing syncytial cells, pale nuclei, slight nuclear pleomorphism, negative reticulin and positive glycogen, and high values indicating cases showing the opposite features. While the overall scores, which ranged from −56 to +128, are distributed throughout the range of observed values, there appears to be some concentration of cases towards each end of the scale. The features of the low-score cases are those usually regarded as characteristic of Ewing's sarcoma, while those of the high-score cases are

FIG. 11.—Distribution of overall scores (T2+T3+T5−T1−T6) for the 40 cases studied.

those usually regarded as characteristic of reticulum-cell sarcoma.

*Relationship to clinical features*

It is of interest to see whether there is any relationship between the combined histological score for a case and other features such as age, sex, anatomical site and clinical behaviour. The results for the 40 cases show a significant correlation (Spearman's rank correlation coefficient, $r=0 \cdot 4852$, $P<0 \cdot 001$) between combined score and age, but no relation between combined score and sex (Wilcoxon's rank sum test, $z=0 \cdot 55$, $P>0 \cdot 10$).

We isolated two groups of cases at the ends of the scale, a low-score group (combined score $< 20$) and a high-score group (combined score $> 80$) where age, sex, anatomical site and length of survival can be directly compared. There are 25 cases (18 males, 7 females) in the low-score (Ewing) group, with a mean age of 16 years, and 12 cases (9 males, 3 females) in the high-score (reticulum-cell sarcoma) group, with a mean age of 48 years. The age distribution of the cases is shown in Fig. 12. While each group shows about the same degree of predominance of male cases, there is a clear difference in age, as shown by the correlation between age and combined score for the whole group of cases and by the mean ages for the low-score and high-score groups.

FIG. 12.—Age distribution for the 40 cases studied, indicating the "low-score" (Ewing) and "high-score" (reticulum-cell sarcoma) cases.

The general pattern of anatomical distribution appears to be the same in the low-score and high-score groups. The majority of tumours involve the long bones (most commonly femur, tibia and humerus) and ribs and pelvis account for most of the others. The series is not really large enough, however, for a detailed study of anatomical site.

When length of survival is considered, the estimated 3-year survival proportion for the low-score group is less (24%) than in the high-score group (42%). But a comparison of the survival times in the two groups by the logrank test (Peto and Peto, 1972) shows that this difference is well within the limit expected ($\chi^2=1 \cdot 59$ on 1 d.f., $P>0 \cdot 10$). It should be appreciated, however, that some of our cases had been collected as part of a study of bone tumour mortality (Boyd *et al.*, 1969), and the fact that surviving patients were excluded from this study may have obscured any differences in the length of survival of the different types of tumour.

*Accuracy and consistency of observers*

Results for the accuracy and consistency of the observations of the 5 pathologists who took part in the study are shown in Tables VIII and IX. A reader's accuracy is judged by a comparison of his results with the mean values of all the readers: his consistency expresses the variation of his repeated observations on the same section. A low value indicates a high degree of accuracy or consistency.

The ranking of the different observers for accuracy and consistency depends, to some extent, on the particular histological feature concerned. Thus while Reader 2 is generally more accurate than Reader 1, this is not the case for Features 4 or 6. Similarly, while Reader 1 is generally more

TABLE VIII.—*Accuracy Scores for the 5 Readers for Each of the 6 Histological Features Studied*

| | Reader | | | | |
|---|---|---|---|---|---|
| Feature | 1 | 2 | 3 | 4 | 5 |
| 1 | 40·0 | 37·7 | 34·5 | 25·3 | 37·0 |
| 2 | 38·1 | 27·1 | 32·4 | 37·9 | 29·9 |
| 3 | 24·8 | 18·3 | 39·9 | 21·7 | 22·5 |
| 4 | 40·4 | 44·2 | 30·6 | 43·6 | 38·0 |
| 5 | 16·1 | 12·6 | 18·3 | 26·9 | 18·5 |
| 6 | 16·6 | 18·6 | 17·2 | 18·8 | 29·2 |

A low value indicates a high degree of accuracy. Values for Features 5 and 6 cannot be compared with those for Features 1–4 but can be compared with each other.

TABLE IX.—*Consistency Scores for the 5 Readers for Each of the 6 Histological Features Studied*

| | Reader | | | | | |
|---|---|---|---|---|---|---|
| Feature | 1 | 2 | 3 | 4 | 5 | Total |
| 1 | 9·0 | 11·5 | 10·5 | 16·5 | 32·5 | 80·0 |
| 2 | 10·0 | 11·0 | 16·0 | 19·5 | 33·5 | 90·0 |
| 3 | 2·5 | 6·0 | 15·0 | 19·5 | 25·0 | 68·0 |
| 4 | 30·0 | 29·0 | 38·0 | 10·0 | 36·0 | 143·0 |
| 5 | 6·7 | 11·5 | 16·0 | 23·3 | 28·0 | 85·5 |
| 6 | 12·7 | 7·3 | 17·3 | 14·7 | 34·7 | 86·7 |
| Total | 70·9 | 76·3 | 112·8 | 103·5 | 189·7 | |

A low value indicates a high degree of consistency.

consistent than Reader 2, this is again not the case for Features 4 or 6. Reader 5 is generally less consistent than the others, although not different from them in accuracy.

The accuracy and consistency scores can also be considered feature by feature. Feature 3, for example, has generally lower values for consistency than the others, although not for all readers, while Feature 4 has generally higher values. The accuracy scores can be compared over the first 4 features: again Feature 3 has generally low values and Feature 4 generally high values. Although the accuracy scores for Features 5 and 6 cannot be compared with those for Features 1–4, it would seem, from the estimates of magnitude of the component R in the analysis of variance, that Features 5 and 6 led to less disagreement among the readers than the other questions.

### DISCUSSION

Very little work appears to have been carried out on the factors responsible for differences of observation and interpretation between pathologists in connection with histological diagnosis, despite the frequency of observer error studies in other fields of medicine. Among the very few published studies is that of Garceau (1964) on the consistency of histological diagnosis of cirrhosis, that of Cocker, Fox and Langley (1968) on the consistency of histological diagnosis of epithelial abnormalities of the cervix uteri, that of Iversen and Sadnes (1971) on the reliability of pathologists in the diagnosis of lymph node biopsy specimens, and that of Lambourne and Lederer (1973) on observer variation in cytological screening for cervical carcinoma.

There are, however, many aspects of histological diagnosis of tumours which would lend themselves to this type of study, an obvious example being the numerical system of grading introduced by Broders (1926) and still applied—often uncritically—to many types of tumour.

In the present study we have been able to establish certain histological differences in a group of round-cell tumours of bone, and also to show the importance of differences between different pathologists, and between the repeated observations of individual pathologists, in recording what initially appeared to be fairly simple histological features of this type of tumour. The method of repeated observation, together with analysis of variance, proved to be an informative one, and would appear to be applicable to many other areas of histological diagnosis, although the significance-testing aspect of the method should not be over-played. In retrospect, our experimental design could have been improved. A larger and less selected group of tumours would clearly have been desirable, but was not available. It would also have been of interest to make observations on duplicate sections stained for reticulin and for glycogen. While the observers made their repeated observations without knowing which cases they were studying, the different questions with regard to a particular case were answered at the same time. It is possible, therefore, that bias due to preconceived associations or dissociations between the different histological features could have entered the study in this way. In future work of this sort it is clear that experimental design needs very careful consideration, particularly at the outset of the study.

The first of our histological features was cell outline, the arrangement of the tumour cells in a syncytial or separate form. The difficulties encountered in making this distinction were partly explained by the presence of both types of cell in many of the tumours, although the study brought to light real differences between tumours, and real differences in the way that the individual pathologists reacted to the problem. Some pathologists showed a bias towards one or other type of cell, or to recording an observation as "indeterminate", and individual pathologists did not necessarily find the same cases "typical" or "indeterminate" as far as their observations were concerned. Caution is clearly needed by any pathologist, however experienced in this field, in reaching a conclusion about this aspect of a round-cell tumour of bone, particularly on the basis of the examination of one section from one block of tissue.

The results for nuclear staining are much the same as for cell outline. Tumours may show both pale and dark nuclei as well as those which are hard to identify as either. The results for nuclear pleomorphism are also similar, although there was less inconsistency among individual readers on this question.

Conspicuousness of nucleoli was a feature which, although it showed significant differences between cases, did not correlate with other features, or help to distinguish between different types of round-cell tumour. There was also much inconsistency and disagreement over the scoring of this feature, and little attention was therefore paid to it in the later part of our study.

The fifth of our histological features was reticulin pattern. There was better agreement between the different pathologists as to whether or not a positive reticulin pattern was present than with the previous histological features, and there was a greater degree of separation of the scores of individual cases into two groups. The same comments can be made with regard to intracellular glycogen.

The results of the present study support the idea that round-cell tumours of bone are heterogeneous for histological structure. The tumours differ from one another for each of the histological features studied, although observer variation and other factors tend to obscure this. Despite this heterogeneity, it is not possible to distinguish completely distinct sub-groups, either on the basis of a single histological feature or a combination of these features. There is, however, a significant correlation between most of the features studied, and used together they allow a particular tumour to be placed on a scoring scale. At one end of the scale are tumours with the histological features commonly regarded as those of Ewing's sarcoma, and at the other are tumours with the features commonly regarded as those of reticulum-cell sarcoma. In between, there are a significant number of tumours which are not typical of either.

It is not possible to decide, from our results, whether the histological heterogeneity of malignant round-cell tumours results from the existence of two or more sub-groups, or from the continuous variation of one basically related group. When other features of the cases designated as "low-score" and "high score" are studied, they appear to differ in age and possibly in length of survival, although the anatomical distribution of the two types of tumours appears similar.

Our results are consistent with the prevailing impression of most pathologists, that an attempt should be made to distinguish histologically, as a diagnostic and prognostic procedure, between the two types of round-cell tumour, but they emphasize the difficulties that can be encountered, at least with the histological features that we selected for study.

The present study does not deal with the question of the histogenesis of these tumours, or their relationship to other types of tumour. It does not help to decide whether all these tumours are of lymphoid origin, nor does it cast any light on Ewing's original concept of endothelial myeloma. The observed features of our high-score cases exist irrespective of whether they are regarded as reticulum-cell sarcoma or histiocytic lymphoma (see Rappaport, 1966) or whatever.

It is unlikely that round-cell tumours of bone are unique in showing, as a group, a marked degree of variation in the various histological features which can be identified for study: indeed, such variation would appear to be a feature of many types of tumour, and objective quantitative studies may well have an important role in establishing the criteria which can be used for their histological subdivision.

An interesting aspect of the study was the insight it provided into the behaviour of the 5 pathologists taking part. We set out with the impression that we were studying round-cell tumours, but ended with the realization that we, ourselves, were equally under scrutiny. Differences between the readers contributed significantly to the total variation and, in this respect, the results of our study are rather similar to what has been found in investigations of differences between examiners in marking various types of paper, including those of the final examination in medicine (Hartog and Rhodes, 1936; Bull, 1956). Differences in accuracy and consistency between the 5 pathologists taking part in the study were also found. Recognition of this subjective element should lead to a more precise definition and understanding of the nature of the histological features being studied, and to some extent this was achieved in the present study. The observers recognized, for example, the error of recording PAS-positive granules in macrophages as "intracellular glycogen", althrough they were not all aware of this at the outset. Similarly, they became aware of the need to record a positive reticulin pattern or a positive result for glycogen even when this involved only a small part of the tissue available for study. The realization, in the later part of the study, that observations on cell outline or nuclear staining recorded what was often only the predominant aspect of a variable appearance, led to a better understanding of these aspects of the cytology of malignant round-cell tumours. Indeed, one of the most important aspects of the observer error study was that it forcibly drew the attention of the observers to points, either in connection with the pathology of round-cell tumours, or in connection with their own attitudes, of which they were not previously aware.

The study was carried out at the suggestion of the Cancer Research Campaign's Bone Tumour Panel, of which the members were, at the time the work started, Prof. R. W. Scarff, Chairman, Dr J. Ball, Dr P. D. Byers, Dr Mary Catto, Dr W. Goldie, and Prof. H. A. Sissons, Secretary. It involved the active participation of the 5 members who acted as observers for the study (Dr Ball, Dr Byers, Dr Catto, Dr Price and Prof. Sissons).

Our thanks are due to Mrs Maureen Cohen, who coped with the complexities of circulating the histological material and collating the results of the 5 observers, to Miss Molly Ng, who typed the many drafts of the paper, and to Mr Terry Davies, who helped to prepare the illustrations.

### REFERENCES

BALL, J. (1970) Ewing's Tumour and Reticulum-cell Sarcoma. In *Symposium Ossium*. Ed. A. M. Jelliffe and B. Strickland. Edinburgh & London: Livingstone.

BOYD, J. T., DOLL, R., HILL, G. B. & SISSONS, H. A. (1969) Mortality from Primary Tumours of Bone in England and Wales, 1961–63. *Brit. J. prev. soc. Med.*, **23**, 12.

BRODERS, A. C. (1926) Carcinoma. Grading and Practical Application. *Arch. Path.*, **2**, 376.

BULL, G. M. (1956) An Examination of the Final Examination in Medicine. *Lancet*, ii, 368.

COCKER, J., FOX, H. & LANGLEY, F. A. (1968) Consistency in the Histological Diagnosis of Epithelial Abnormalities of the Cervix Uteri. *J. clin. Path.*, **21**, 67.

DAHLIN, D. C. (1965) Ewing's Sarcoma and Malignant Lymphoma (Reticulum-cell Sarcoma) of Bone. In *Tumors of Bone and Soft Tissue*. Chicago: Year Book Medical Publishers.

DAHLIN, D. C., COVENTRY, M. D. & SCANLON, P. W (1961) Ewing's Sarcoma. A Critical Analysis of 165 Cases. *J. Bone Jt. Surg.*, **43A**, 185.

FRIEDMAN, B. & HANAOKA, H. (1971) Round-cell Sarcoma of Bone. A Light and Electron Microscopic Study. *J. Bone Jt. Surg.*, **53A**, 1118.

GARCEAU, A. J. (1964) The Natural History of Cirrhosis. *New Engl. J. Med.*, **271**, 1173.

HARTOG, P. & RHODES, E. C. (1936) *The Marks of Examiners*. London: Macmillan.

IVERSEN, O. H. & SADNES, K. (1971) The Reliability of Pathologists. A Study of Some Cases of Lymph Node Biopsies Showing Giant Follicular Hyperplasia or Lymphoma. *Acta path. microbiol. scand.*, **79**, 330.

JOHNSON, N. L. & LEONE, F. C. (1964) *Statistics and Experimental Design in Engineering and the Physical Sciences*. Vol. II, Chapter 13. New York: John Wiley.

LAMBOURNE, A. & LEDERER, H. (1973) Effects of Observer Variation in Population Screening for Cervical Carcinoma. *J. clin. Path.*, **26**, 564.

LICHTENSTEIN, L. & JAFFE, H. L. (1947) Ewing's Sarcoma of Bone. *Am. J. Path.*, **23**, 43.

LUMB, G. & MACKENZIE, D. H. (1956) Round-cell Tumours of Bone. *Br. J. Surg.*, **43**, 380.

PARKER, F. & JACKSON, H. (1939) Primary Reticulum-cell Sarcoma of Bone. *Surgery Gynec. Obstet.*, **68**, 45.

PETO, R. & PETO, J. (1972) Asymptotically Efficient Rank Invariant Test Procedures. *Jl R. statist. Soc.* (Series A), **135**, 185.

PRICE, C. H. G. (1973) A Critique of Ewing's Tumour of Bone. In *Bone: Certain aspects of Neoplasia*. Ed. C. H. G. Price & F. G. M. Ross. London: Butterworths. p. 177.

RAPPAPORT, H. (1966) Tumors of the Haematopoietic System. In *Atlas of Tumor Pathology*. Section III, Fascicle 8. Washington: A.F.I.P.

SCHAJOWICZ, F. (1959) Ewing's Sarcoma and Reticulum-cell Sarcoma of Bone, with Special Reference to the Histochemical Demonstration of Glycogen as an Aid to Differential Diagnosis. *J. Bone Jt. Surg.*, **41A**, 349.

SEARLE, S. (1971) Topics in Component Variance Estimation. *Biometrics*, **27**, 1.

STOUT, A. P. (1943) A Discussion of the Pathology and Histogenesis of Ewing's Tumor of Bone Marrow. *Am. J. Roentg.*, **50**, 334.

WILLIS, R. A. (1940) Metastatic Neuroblastoma in Bone Presenting the Ewing's Syndrome with a Discussion of "Ewing's Sarcoma". *Am. J. Path.* **16**, 317.